# THEMS: an automated thermal and hyperspectral proximal sensing system for canopy reflectance, radiance and temperature

**DOI:** 10.1186/s13007-020-00646-w

**Published:** 2020-07-31

**Authors:** William Woodgate, Eva van Gorsel, Dale Hughes, Lola Suarez, Jose Jimenez-Berni, Alex Held

**Affiliations:** 1 Commonwealth Scientific and Industrial Research Organisation, CSIRO, Building 801, Black Mountain, ACT 2601 Australia; 2 School of Earth and Environmental Sciences, The University of Queensland, Brisbane, QLD 4067 Australia; 3 Fenner School of Environment and Society, Australian National University, Acton, ACT 2601 Australia; 4 Department of Infrastructure Engineering, The University of Melbourne, Parkville, VIC 3010 Australia; 5 School of Agriculture and Food. Faculty of Veterinary and Agricultural Sciences, The University of Melbourne, Parkville, VIC 3010 Australia; 6 Instituto de Agricultura Sostenible (IAS), Consejo Superior de Investigaciones Cientificas (CSIC) Avenida Menéndez Pidal, Campus Alameda del Obispo, 14004 Córdoba, Spain

**Keywords:** THEMS, Hyperspectral, Thermal, Remote sensing, Proximal sensor, Validation, Flux tower, Tumbarumba

## Abstract

**Background:**

Earth Observation ‘EO’ remote sensing technology development enables original insights into vegetation function and health at ever finer temporal, spectral and spatial resolution. Research sites equipped with monitoring infrastructure such as flux towers operate at a key bridging scale between satellite platform measurements and on-the-ground leaf-level processes.

**Results:**

This paper presents the technical details of the design and operation of a proximal observation system ‘THEMS’ that generates unattended long-term high quality thermal and hyperspectral images of a forest canopy on a short (sub-daily) timescale. The primary purpose of the system is to measure canopy temperature, spectral reflectance and radiance coincident with a highly instrumented flux tower site for benchmarking purposes. Basic system capability is demonstrated through low level data product descriptions of the high-resolution multi-angular imagery and ancillary data streams. The system has been successfully operational for more than 2 years with little to no intervention.

**Conclusions:**

These data can then be used to derive remotely sensed proxies of canopy and ecosystem function to study temporal forest dynamics over a wide range of wavelengths, spatial scales (individual trees to canopy), and temporal scales (minutes to multiple years). The multi-purpose system is intended to provide unprecedented spatio-temporal ecophysiological insight and to underpin upscaling of remotely sensed dynamic ecosystem water, CO_2_, and energy exchange processes.

## Background

Bridging temporal and spatial scales for monitoring the state and function of vegetation across the terrestrial biosphere remains a challenge [18, 55]. Earth observation (EO) remote sensing applies the principles of vegetation spectroscopy to study the interrelationships between the spectral characteristics of vegetation and their biophysical attributes from the leaf level through to the global scale [2, 25, 29].

A number of regional and global research networks and collaborations have formed in recognition of this challenge, including: SpecNet [23] a network of near-surface remote sensing platforms co-located with terrestrial flux tower sites measuring the instantaneous rate of exchange of carbon and water fluxes between the canopy and the atmosphere [7], the PhenoCam network [10, 58] comprising automated digital time‐lapse cameras or ‘phenocams’ to monitor vegetation status and environmental changes over long periods of time ‘phenology’, and the SENSECO, OPTIMISE and EUROSPEC European Cooperation in Science and Technology ‘COST’ actions (CA1734, ES1309 and ES0903, respectively) to support innovative optical or spectral sampling tools for proximal sensing of ecophysiological processes. The focal point of each of these initiatives is near-ground or proximal remote sensing platforms, such as unmanned aerial vehicles (UAVs) or tower-mounted platforms pointing down at the canopy.

Proximal systems greatly complement satellite EO products with their increased spatial, spectral, and temporal sensor resolution. Specifically, these multi-purpose optical systems can serve to: validate base-level satellite products (e.g. top-of-canopy reflectance, radiance, or temperature), verify higher level products (e.g. vegetation phenology, Leaf Area Index, solar-induced chlorophyll fluorescence ‘SIF’), and up-scale dynamic vegetation functioning processes to bridge the leaf-to-canopy scale continuum [33]. These systems can also complement flux tower data partitioning and gap-filling procedures [18], which are subject to high levels of uncertainty and strict data filtering requirements [57, 67]. Such systems have the advantage over UAV systems as being ideally suited for frequent and long-term unattended monitoring, which complement long-term research sites with established infrastructure providing insights into ecosystem dynamics across time scales [1].

Technology advancements have been a key driver of proximal system development enabling new applications at co-located flux tower sites. There has been a considerable increase in spectral and spatial resolutions of commercially available sensors since the pioneering applications of the UniSpec spectrometers (PP Systems, MA, USA) by Gamon et al. [20] and Leuning et al. [47]. These systems were deployed for reflectance monitoring of groundcover and canopy dynamics from ground and tower platforms, respectively. More recently, multiple optical sensors were combined to enhance the measured spectral resolution (band width and spectral range) for combined applications. For example, Meroni et al. [51] combined a two spectrometers, one covering the visible and near-infrared (VNIR) region to extract multiple spectral indices, and the second high spectral resolution spectrometer operating in the NIR to extract the sun-induced chlorophyll fluorescence signal. Combining these two types of spectrometers is now becoming commonplace [12, 14, 60]. Aubrecht et al. [5] deployed a high spatial resolution thermal imager providing unprecedented temporal and spatial insights into the Harvard forest thermal regime, however this was mounted at a stationary fixed angle. Accordingly, Gamon et al. [18] argued there was ample opportunity for further exploration and clarification of diurnal to seasonal plant function drivers by integrating indices across spectral domains from visible to thermal wavelengths at highly instrumented flux tower sites.

The paper presents the technical details of the design and operation of a proximal observation system ‘THEMS’ that generates high quality thermal and hyperspectral images of a forest canopy on a short (sub-daily) timescale. Low level data products are also presented to demonstrate basic system capability. The primary purpose of the system is to measure canopy temperature, spectral reflectance and radiance coincident with a highly instrumented flux tower site for benchmarking purposes. These data can then be used to derive remotely sensed proxies of canopy and ecosystem function to study temporal forest dynamics over a wide range of wavelengths, spatial scales (individual trees to canopy), and temporal scales (minutes to multiple years).

The structure of the manuscript is as follows: first, the Tumbarumba study site is described followed by a description of the THEMS hardware, software and measurements acquired. Next, the data processing, data filtering, and validation steps are outlined. Basic data products and system capabilities are then demonstrated in the Results section. Finally, the importance and challenges associated with combining high spatial-, temporal-, and spectral-resolutions are then discussed followed by the concluding remarks.

## Materials and methods

### Site description

The *Tumbarumba Hyperspectral and thErmal Monitoring System* ‘*THEMS*’, with the capitalised letters denoting the naming origin, is located 68 m above ground at the top of the Tumbarumba flux tower, New South Wales, Australia (35° 39′ 20″ S, 148° 09′ 07″ E, 1200 m elevation). The site was established in 2001 and is a member of FLUXNET [7], SPECNET [23], and part of the Australian Terrestrial Ecosystem Research Network (TERN; [8, 41]). The site is characterised by a moderately open wet sclerophyll forest, with a 40 m tall canopy and dominant overstory species of *Eucalyptus delegatensis* and *Eucalyptus dalrympleana* [43, 46].

### THEMS system description

**Fig. 1:**
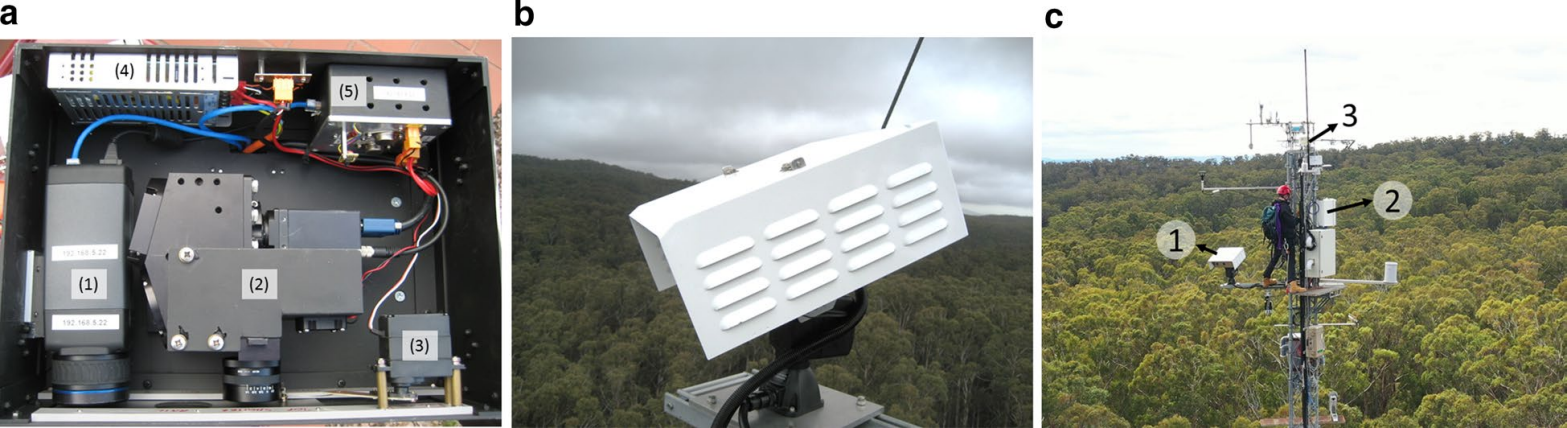
**a** Internal view of the main sensor enclosure. (1) thermal camera, (2) VNIR hyperspectral line scanner with cooling fan, (3) shutter motor and mechanism, (4) 24 VDC to 12 VDC converter, and (5) shutter controller with temperature sensor. **b** External view of the main sensor enclosure on the pan tilt unit, tilted downward at the canopy. The white cover is the radiation shield with ventilation. **c** Contextual view of the THEMS sensor enclosure (number ‘1’ insert). Other inserted numbers are: 2) the command PC ensclosure, and 3) the all sky camera and irradiance sensor location

A main feature of the system is the co-location of a visible and near-infrared ‘VNIR’ hyperspectral line scanner ‘HLS’ (model VNIR N-Series, Headwall Photonics Inc., MA, USA), and the thermal camera (model A655SC, FLIR Systems Inc.) in the main sensor enclosure. The sensors are mounted in a custom-built enclosure attached to a pan tilt unit ‘PTU’ (model PTU-D48E, FLIR Systems Inc.) for multi-angular acquisition (Fig. 1a, b). These sensors acquire multi-angular observations of spatially resolved canopy radiance (W m^−2^ sr^−1^ µm^−1^) and temperature (K). Two other main system sensor components are the: (i) a VNIR spectrometer (model USB 2000+, Ocean Insight Inc.) attached to a cosine diffuser to measure down-welling hemispherical irradiance (W m^−2^ µm^−1^), and (ii) a hemispherical camera (model Q25, Mobotix, Germany) to capture an image of the sky conditions. Table 1 outlines the main sensor specifications in more detail. The radiation shield combined with the cooling fan helps stabilise the temperature of the sensors in the main sensor enclosure (Fig. 1a, b). Also, the enclosure incorporates a mechanical shutter operated with a servo motor (Fig. 1a) that opens and closes the optical windows for the cameras. This shutter also allows the acquisition of dark images to perform the dark current calibration for the radiometric calibration.

**Table 1:** THEMS four main sensor specifications

Sensor	Spatial resolution (pix)	FOV (deg)	Wavelength range (µm)	Spectral bands (#)
VNIR HLS (radiance)	1004 × 1	26.58 θ, 0.03 φ	0.4–1	1004
Thermal camera	480 × 640	18.75 θ, 25 φ	7.5–14	1
VNIR spectrometer (irradiance)	1 × 1	180 θ φ	0.2–1.1	2048
Hemispherical camera	3072 × 2048	180 θ φ	0.4–1	3

*Pix* pixels, *FOV* angular instrument field of view (FOV) in degrees (θ = elevation angle, φ = azimuth angle)

**Fig. 2:**
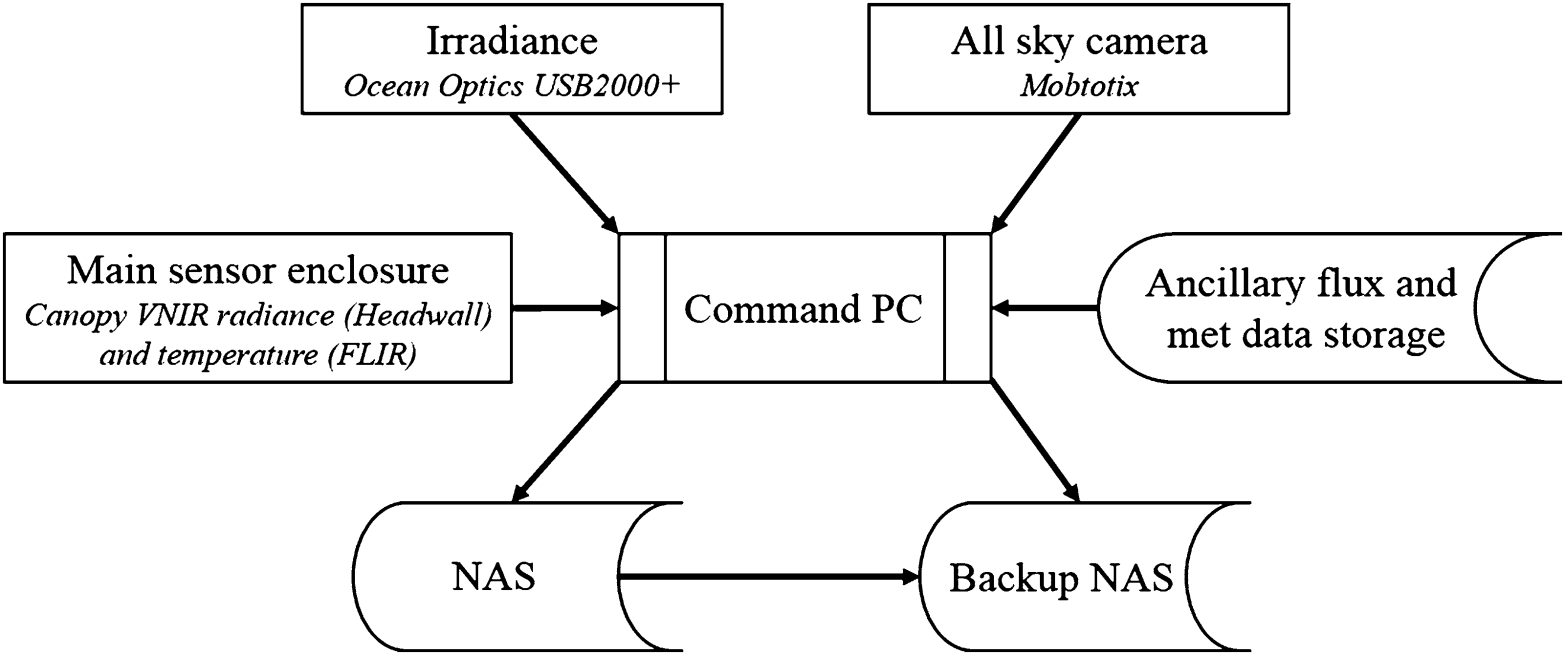
Schematic of main system components. Arrows represent the direction of data transfer. The ancillary flux and meteorological data transferred to the command PC include sky temperature, ambient air temperature, and relative humidity. The main sensor enclosure houses the FLIR thermal camera and the Headwall VNIR hyperspectral line scanner ‘HLS’ (Fig. 1a)

Ancillary sensors collect measurements that are required to correct for thermal camera interferences, described in more detail in “Data collection” section. These measurements include an ambient air temperature and relative humidity probe (HMP 50, Vaisala Inc.), a pyrgeometer (CNR4, Kipp and Zonen) for sky temperature and incoming shortwave radiation, and a sunshine sensor (BF5, Delta-T Inc.) for measuring incoming PAR radiation. These measurements are stored by a Campbell Scientific CR3000 data logger as part of the co-located flux measurement system and then retrieved by the central command PC and stored alongside the radiance and irradiance data. The command PC transfers data acquisitions via Ethernet to two Network Attached Storage (NAS) devices located in an enclosure at the base of the tower; one as primary storage and the second as a backup, which stores a copy of the data in a compressed form. A schematic of the main system components is depicted in Fig. 2.

The command PC and irradiance sensor are in an enclosure near the PTU and main sensor enclosure. The command PC is a ruggedized industrial PC with an additional cooling fan installed to cool the main heatsink. The input to the irradiance sensor is via an optical fibre which goes to the top of the tower for the down-welling hemispherical irradiance measurement. The system is powered by the site’s main power supply consisting of a battery bank connected to a diesel generator, located on the ground level.

Pencil style Argon and Xenon spectral calibration lamps (Oriel, model 6030 and 6033) were used to verify band centres of the irradiance sensor and to define the correspondence of the spectral pixels and the wavelengths in the HLS. The radiometric calibration of the irradiance sensor and hyperspectral line scanner camera was undertaken using a uniform radiance source (HELIOS 20” D-Series Uniform Light Source Dynamic Range System, Labsphere Inc.) composed of an integrating sphere and four independent light sources (1 × 150 W lamp, 3 × 35 W lamps) that provide different levels of known spectral radiance. Both instruments displayed a high degree of linearity when varying lamp intensity (number of lamps and manual shutter for a single lamp) and when varying integration time and gain. Calibration functions were then calculated using the reference lamp intensity provided in the Luminance Calibration Certificate of the uniform radiance source.

Thermal camera validation was undertaken in a temperature controlled room using two blackbody instruments. The first instrument was a HyLogger-3 unit [63] consisting of two temperature controlled blackbody targets at 20 °C and 60 °C, respectively, with 0.98–1 emissivity over the 5–15 µm spectral range. The second blackbody was a custom-made brass container partially filled with water designed to minimise light entering and escaping. Both blackbody instrument results agreed to within ± 0.5 °C of the FLIR sensor output, notably within the ± 0.5 °C estimated uncertainty of the HyLogger-3 (L Whitbourn 2018, pers. comm., 3 August).

### Data collection

The controlling software that runs the system on the command PC was developed in LabVIEW 2013 (National Instruments Corporation, Austin, TX, USA) integrated with a FLIR LabVIEW software development kit and Dynamic Link Libraries from Ocean Insight Inc. The software consists of two main modules: (i) THEMS, which performs instrument control, positioning and data acquisition, and (ii) SuperTHEMS, which acts as a system supervisor and initialises at PC start-up to control THEMS, it provides data handling services to THEMS at the end of each day (see Additional file 1 for an image of the controller software GUIs).

The software is designed to run autonomously and continuously with little or no user intervention once the system has been configured. Extensive context sensitive help is available on all THEMS settings and more detailed online help is available for many functions and controls, available upon request. A site cellular phone tower network connection enables remote PC access and manual intervention if required. The controlling LabVIEW source code can be made available upon request for academic applications.

The system is primarily triggered to acquire data based on hour of day or solar elevation angle given the site geographic coordinates and an input reference elevation angle. From 2015 to 2018 acquisitions were always made at solar noon, while acquisitions at the predefined reference solar elevation angle are recorded if the times at which the reference solar elevation occur differs by more than a selectable time interval; the default interval is 20 min. The default reference angle at the Tumbarumba site is the solar elevation at solar noon on mid-winter’s day (June 21st—winter solstice), ensuring data acquisition all year round. Apart from a few days around June 21st the sun will pass through this reference elevation twice each day; before and after solar noon. Other acquisition mode options include: autonomous capture before sunrise and just after sunset, every hour, or on-demand via remote access.

**Fig. 3:**
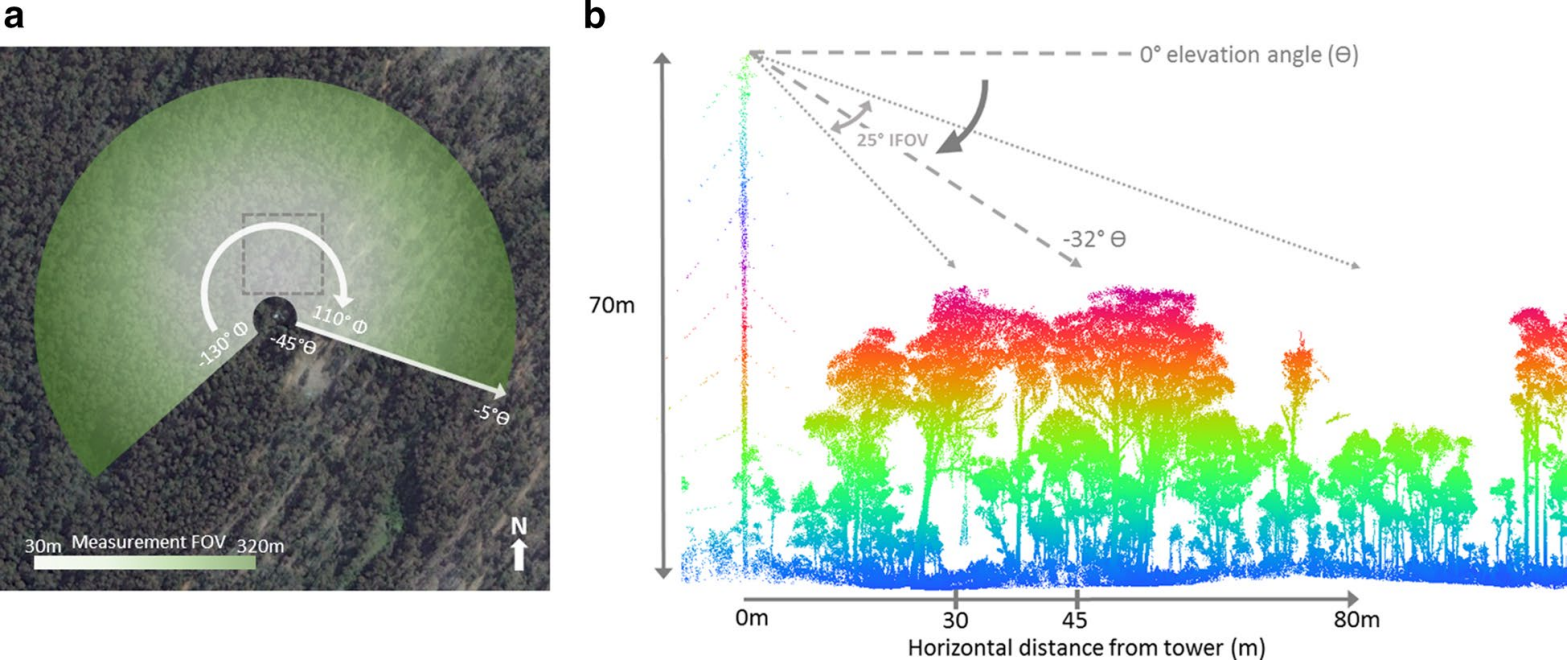
**a** Birds-eye view of the projected measurement field-of-view (FOV) from two elevation scans. The flux tower is located at the image centre. The FOV is calculated as the top of canopy projected area when conducting two elevation scans (θ) at − 20° and − 32° (θ range from − 45° to − 7.5°), starting at − 130° azimuth (φ) through to 110° denoted by a white circular arrow in the clockwise direction. The projected area starts 30 m from the tower location and finishes at range of around 320 m, with some elevation angle overlap (≈ 12֯) between the two scans. The dashed grey square denotes the 1 ha TERN SuperSite plot [41]. Image from Google Earth Pro (image date: 14/4/2016). **b** Profile view schematic of the − 32° elevation acquisition FOV overlaid on a point cloud slice coloured by height, acquired May 2016 using a Riegl VZ400 terrestrial laser scanner. The 70 m flux tower is visible on the left-hand side of figure, with THEMS sensors located at the top. The dominant trees are around 40 m tall

Two panoramic azimuthal scans at different elevation angles are completed for each triggered acquisition in order to cover a greater sample area, equating to around 21 ha of projected top of canopy area (Fig. 3). The central elevation angles are nominally -20° and − 32.5°, equating to a zenith range of around 40° (− 7.5° to − 45° zenith with ~ 12° of overlap between scan angles). The pan-tilt unit covers an azimuth range of around 240° (− 130 to 110° azimuth; Fig. 3), enabling an image panorama to be acquired. The time taken to complete one elevation angle acquisition is around 5 min, with the rotational speed determined by the need to achieve the best hyperspectral image aspect ratio. Irradiance and sky image measurements are acquired at the beginning and end of each acquisition (i.e. each pair of elevation angle measurements will lead to four irradiance and four sky camera measurements). A recent revision to the software allows irradiance measurement acquisition approximately every second while the PTU is moving.

A key difference between the thermal and hyperspectral imaging sensors is that the former is a 2D imager (480 × 640 pixels), where the latter is a line scanner (1004 × 1 pixels), which must be moved to generate 2D panoramic images. The hyperspectral camera operates at 25 Hz to produce a complete azimuthal image panorama, whereas the thermal camera produces an image stack or video at 6.25 Hz, which includes > 90% image overlap. In the vertical axis there is marginally less spatial overlap of the thermal camera compared to the hyperspectral scanner (18.7° versus 26.6° FOV) when operating at the − 20° and − 32.5° elevation angles due to a slightly smaller thermal sensor FOV (Table 1).

The large data volume produced by the hyperspectral and thermal sensors presents a trade-off in frequency of acquisition, especially as the system is intended for a long-term (> 1 year) unattended monitoring. Each hyperspectral and thermal acquisition consisting of two elevation angle scans produces 31 GB and 5 GB of data, respectively. This equates to around 40 TB year^−1^ when operated three times per day, and meant that on average a field visit every 2 months occured to swap the NAS devices.

The system was installed at Tumbarumba in June 2015 and has been operating near continuously until February 2018 when it was taken down for re-calibration. The system has only missed acquiring data on 53 days out of 950 days to February 2018. It was re-installed in February 2019 and re-configured to collect hourly thermal imagery day and night until it was removed in April 2019 for further development.

Special care was given to the data handling and transfer procedure to ensure no tampering. The primary NAS for data storage is security tagged and located in a locked room on site. When the NAS is ready to be swapped, the security tag is checked for tampering and the primary NAS is replaced with the secondary NAS, which is then tagged and locked up. The NAS removed from the site is then relocated to a data ingestion room where the data is transferred via a secure network connection to the CSIRO Bowen Research Cloud facility. MD5 check sums are completed to ensure data integrity. If there is data corruption or evidence of tampering, the data is considered lost and discarded.

Ancillary terrestrial LiDAR data was acquired with a RIEGL VZ-400 3D terrestrial laser scanner (TLS) from the same position as the THEMS monitoring system on May 18^th^, 2016. This data was used calculate structural metrics used in the data post-processing stages, further explained in subsequent sections. This time-of-flight scanner has a range up to 350 m, 0.35 mrad beam divergence and acquired data at an angular resolution of 0.06°. The multi-return instrument leads to improved vertical sampling compared with single return instruments [48].

### Data format and processing

All the image data streams from the sensors are stored in raw format. The FLIR thermal camera returns single precision 32-bit values and the binary data is stored in little-endian order. The hyperspectral line scanner returns 10-bit data stored as 16-bit unsigned integer values in little-endian order. The processing procedure from raw measurements through to reflectance for the HLS VNIR instrument and canopy temperature corrected for thermal interferences using the thermal camera is described below.

#### Hyperspectral (VNIR) imagery

A dark current correction is applied to the raw DNs of both the HLS and irradiance sensors. In the HLS, 100 frames are collected with the closed shutter. The image is then averaged, which creates a dark current frame that is subtracted from each spatial and spectral pixel. In the case of the irradiance sensor, the spectrometer includes dark pixels that are used for subtracting the dark current as a baseline. Next, sensor-specific calibration files are applied to derive radiance and irradiance from the HLS and irradiance spectrometer, respectively. The irradiance data is then convolved to match the spectral characteristics of the radiance data from the HLS, i.e. band centres and band widths. Hemispherical Conical apparent reflectance, hereafter referred to as reflectance ‘*ρ*’ [nm] calculated using the radiance ‘*L*’ [W m^−2^ sr^−1^] and irradiance data ‘*E*’ [W m^−2^] (Eq. 1) [52, 62]. The start and end irradiance measurements are linearly interpolated to match the dimensions of the VNIR panorama when calculating reflectance.

1 $$\rho \left( \lambda \right) = \pi L\left( \lambda \right)/E\left( \lambda \right)$$

#### Thermal imagery

A thermal panorama is created for each scan elevation angle by extracting the central columns from each frame, determined by frame rate. The data is acquired as total energy reaching the sensor in raw binary format. Object surface temperature is then calculated largely following the method of Aubrecht et al. [5]. In brief, the total energy received at the sensor is corrected for thermal interferences from the air column transmission between the sensor and target caused by water vapour attenuation. The transmission is calculated using the parameters: distance to target (convolved to 1° angular resolution) as measured by a Riegl VZ400 terrestrial laser scanner acquired from the same position as the sensors, water vapour concentration calculated from relative humidity and air temperature, and LOWTRAN atmospheric transmission simulation results stored in the thermal camera image header. The contributions of the air column and reflected objects were subtracted from the total energy received by the sensor. For this work we assumed a constant leaf, bark and ground emissivity of 0.95 and sky emissivity of 1 following Aubrecht et al. [5].

### Data filtering and validation

Data filtering is an optional step applied to discard acquisitions with dense fog or rainfall, and acquisitions during rapidly changing sky and illumination conditions, which lead to unstable reflectance and thermal images. The strictness of data filtering is dependent on the intended application. Four independently collected data streams at the site are proposed to assess the quality of the data acquisition: (i) the irradiance data, (ii) above-canopy 1 Hz PAR data (BF5, Delta-T Inc.), (iii) the all sky camera images used to visualise sky conditions, and (iv) the meteorological data measured by the flux tower. First, the ratio of the beginning and end irradiance measurements can be used as a quantitative indicator of sky stability, for example filtering out all acquisitions with a ratio outside of 1 ± 0.1 range. At Tumbarumba, approximately 50% of acquisitions fall outside this range, with a higher frequency of cloudy conditions in the afternoon. In addition, the coincidently collected PAR data at one second frequency allows a more robust and continuous indicator of sky stability throughout the 5 min scan duration. A combination of quantitative PAR-derived metrics such as mean, standard deviation, minimum, and maximum values can be used to filter out acquisitions, or more simply the ratio of mean to standard deviation is indicative of sky condition stability. The PAR data filtering window can be extended to match the one hourly block averaged fluxes of heat, water vapour and CO_2_ data to ensure comparable sky conditions with the shorter duration optical scans. The PAR data has also been used as an independent check to quantify the temperature sensitivity common to spectrometer systems [34], and in turn can be used to correct first order temperature sensitivity effects by convolving the irradiance data to the same spectral response function as the PAR sensor. Prior to the availability of diffuse radiation measurements at the site from the BF5 pyranometer (from 2017), the clearness index was used as a diffuse sky condition indicator (diffuse fraction = 1 − clearness index) calculated as fraction of actual to potential incoming shortwave radiation.

Additional HLS validation was undertaken in the field using an ASD FieldSpec 3 Pro (ASD Inc.; Boulder, Colorado, USA). Spectral end-members were collected using an ASD spectrometer fitted with a contact probe and a leaf clip attachment in March and May, 2016. ASD spectrometer and HLS foliage measurements were collected from the same *Eucalyptus delegatensis* tree during the same week in March, 2016. Groundcover spectra was also collected with the ASD spectrometer in May 2016 and comprised a mix of leaf litter and grass in representative proportions. The HLS foliage and bark end-members were extracted from the 15:37 acquisition on 20th March, 2016 using the − 32° elevation angle. The HLS groundcover was extracted from the 12:14 acquisition on 8th Jan, 2016 using the − 20° elevation angle. Around 30–50 pixels from a single region were used for each HLS spectra. For the field validation of the temperature observations, ground cover pixels were masked out of the thermal imagery using the co-registered TLS derived canopy mask. Briefly, ground- and canopy-cover masks at 0.5° resolution were generated using a height above ground threshold (0.5 m) so that these two classes could be separately analysed in the thermal imagery.

### Statistical analysis

A quantitative example of the system’s performance is presented, focusing on time series spectral indices and their ability to track 1 year (2017) of daily averaged Gross Primary Production ‘GPP’ CO_2_ fluxes measured by the co-located eddy covariance system. A subset of vegetation indices were calculated that have a demonstrated ability to track photosynthetic capacity at different spatial and temporal scales, namely: the normalised difference vegetation index ‘NDVI’ (R800 − R645)/(R800 + R645) [65], the chlorophyll/carotenoid index ‘CCI’ (R645nm − R531nm)/(R645nm + R531nm) [21], the photochemical reflectance index ‘PRI’ (R531nm − R570nm)/(R531nm + R570nm) [22], and triangular PRI ‘tri-PRI’ (0.5[(520 − 490)(R545nm − R490nm) − (545 − 490)(R520nm − R490nm)] [70]. Mean vegetation index values were used, which produced comparable results to median values (data not shown). Solar noon scans at the − 32° elevation angle were used for the analysis, taking an 80° FOV subset centred on 0° azimuth to minimise BRDF effects. Prior to spectral analysis, the 0.7 nm band spacing reflectance data was smoothed using a 10 nm moving average to increase the signal-to-noise ratio without losing the pigment absorption features. In total, 326 of 365 days were available for analysis due to some power outages, data transfer, and ancillary instrument outages. 1 Hz PAR data (BF5, Delta-T Inc.) was used to determine a sky stability indicator for the 5 min scan duration, calculated as PAR mean/PAR standard deviation, where higher values indicate increased stability. GPP and shortwave down-welling ‘SW↓’ radiation data was averaged daily. Linear models with an intercept were fit using GPP as the dependent variable in python’s scipy stats library. Quality controlled GPP data was calculated following the standard OzFlux network procedures [8].

## Results

**Fig. 4:**
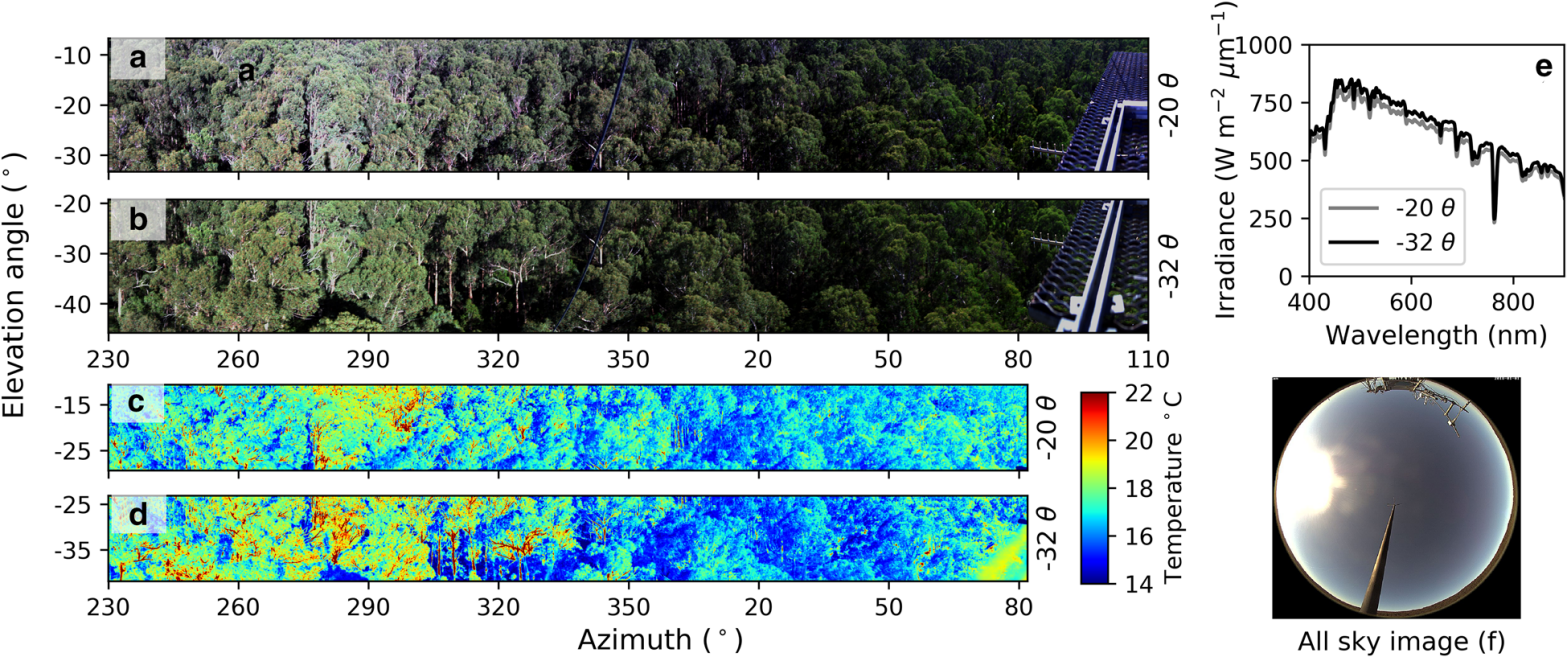
An exemplar of outputs from a single temporal acquisition of the VNIR HLS and thermal camera at two elevation angles for a morning scan on Jan 1st, 2018 at 07:45 (UTC + 10). **a**, **b** depicts RGB true colour composites at the − 20° *θ* and − 32.5° *θ* central elevation scan angles; **c**, **d** depicts the corresponding thermal imagery corrected for thermal interferences with the temperature colour bar to the right hand side; **e** depicts the irradiance from the start of the − 20° *θ* and − 32.5° *θ* central elevation scans; **f** depicts the all sky image from the start of the − 20° *θ* scan. Sun position at time of acquisition: 98° azimuth, 31° elevation. Part of the mounting platform is visible in the right-hand-side of the scans. Note that this and subsequent panoramic images are not orthorectified

An example of the measurements acquired from a single acquisition of the system during clear sky conditions is given in Fig. 4. A typical acquisition consists of simultaneous panoramic measurements using the VNIR HLS (Fig. 4a, b) and thermal cameras (Fig. 4c, d) from 213° to 110° azimuth in a clockwise direction at two elevation angles (− 20° θ and − 32.5° θ elevation, respectively). Irradiance data and sky images (Fig. 4e, f) are collected at the beginning and end of each panoramic scan. A recent modification to the system software sees irradiance measurements collected every few seconds during each scan.

**Fig. 5:**
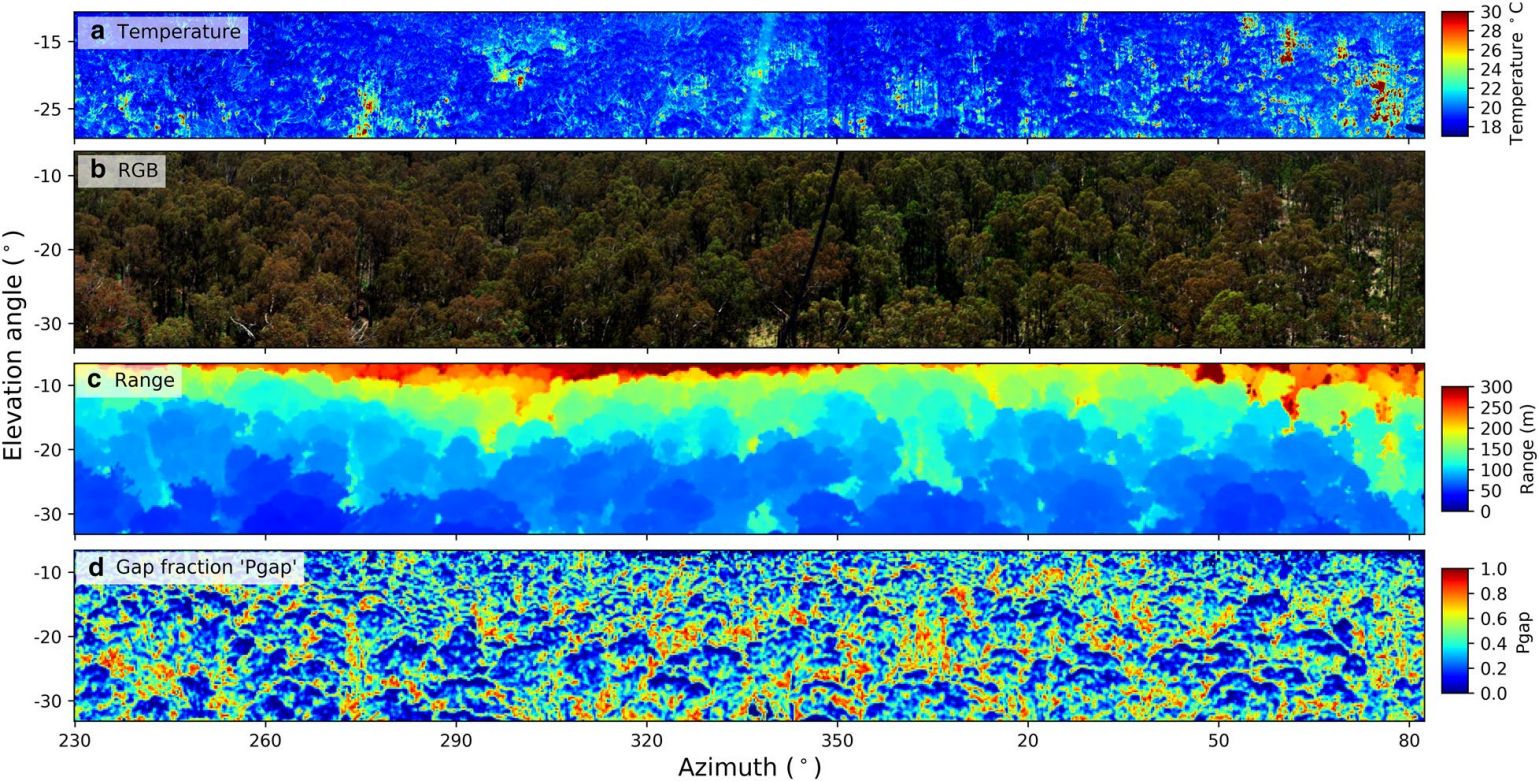
Thermal, hyperspectral and terrestrial laser scanning data panoramas: **a** thermal panorama at the − 20° elevation angle; **b** RGB true colour panorama from the hyperspectral imager; **c** range-to-target image panorama from a Riegl VZ-400 terrestrial laser scanner (TLS), calculated as the distance to the first return for each grid cell (1° × 1° aggregation); **d** Gap fraction ‘Pgap’ or transmissivity image from the TLS, calculated as the fraction of LiDAR first returns beyond 5 m divided by all first returns for each grid cell (1° x 1°). The TLS was acquired from the perspective of the monitoring system May 18th, 2016. The thermal and hyperspectral imagery was from Jan 8th, 2016 at 12:14 (UTC + 10). Sun position at time of acquisition: 0° azimuth, 77° elevation

During solar noon acquisitions much of the background is illuminated due to increased light penetration through the canopy, especially in summer with high solar elevation angles (Fig. 4b). The illuminated regions of the forest floor are up to around 12 °C warmer than foliage under the direct sun conditions in Fig. 4a, creating the largest temperature gradient in the panorama. The within-canopy temperature range of the foliage is approximately 5 °C for the days measured (Figs. 4 and 5). Sunlit branches and stems are around 2–4 °C warmer than sunlit vegetation.

The range to target from the perspective of the thermal and hyperspectral sensors is depicted in Fig. 5. Due to the oblique and multi-angular nature of the imagery the range to the target varies substantially, from around 300 m at shallow elevation angles of − 5° (Fig. 5c) down to 40 m at oblique angles closer to − 45°. This large degree of range variability leads to different magnitudes of atmospheric transmission that needs to be accounted for when computing both canopy temperature [5, 39] and the faint solar induced chlorophyll fluorescence signal [61].

The proportion of the signal passing unobstructed through the canopy, also referred to as ‘gap fraction’ or ‘Pgap’ was calculated using the TLS data (Fig. 5d). The TLS point cloud was aggregated in a 1° × 1° grid. For each cell, Pgap was defined as the fraction of LiDAR first returns beyond 5 m divided by all first returns for each grid cell. This enabled the proportion of the signal coming from top-of-canopy elements versus background components such as soil to be quantified, especially useful for understanding where the spatially resolved thermal and optical signals are originating. For example, much of the sunlit areas from the tops of crowns have a very low transmittance (< 0.1 gap fraction), meaning little background is visible and that near pure canopy pixels are identifiable.

The strong diurnal radiation anisotropy or bidirectional reflectance distribution function ‘BRDF’ effects are clearly visible in the imagery, both for a single image and for different times of day due to different view and sun angle geometries (Fig. 5). There is the strong hotspot effect in the morning acquisition around 278° azimuth, with the least amount of shading [30].

**Fig. 6:**
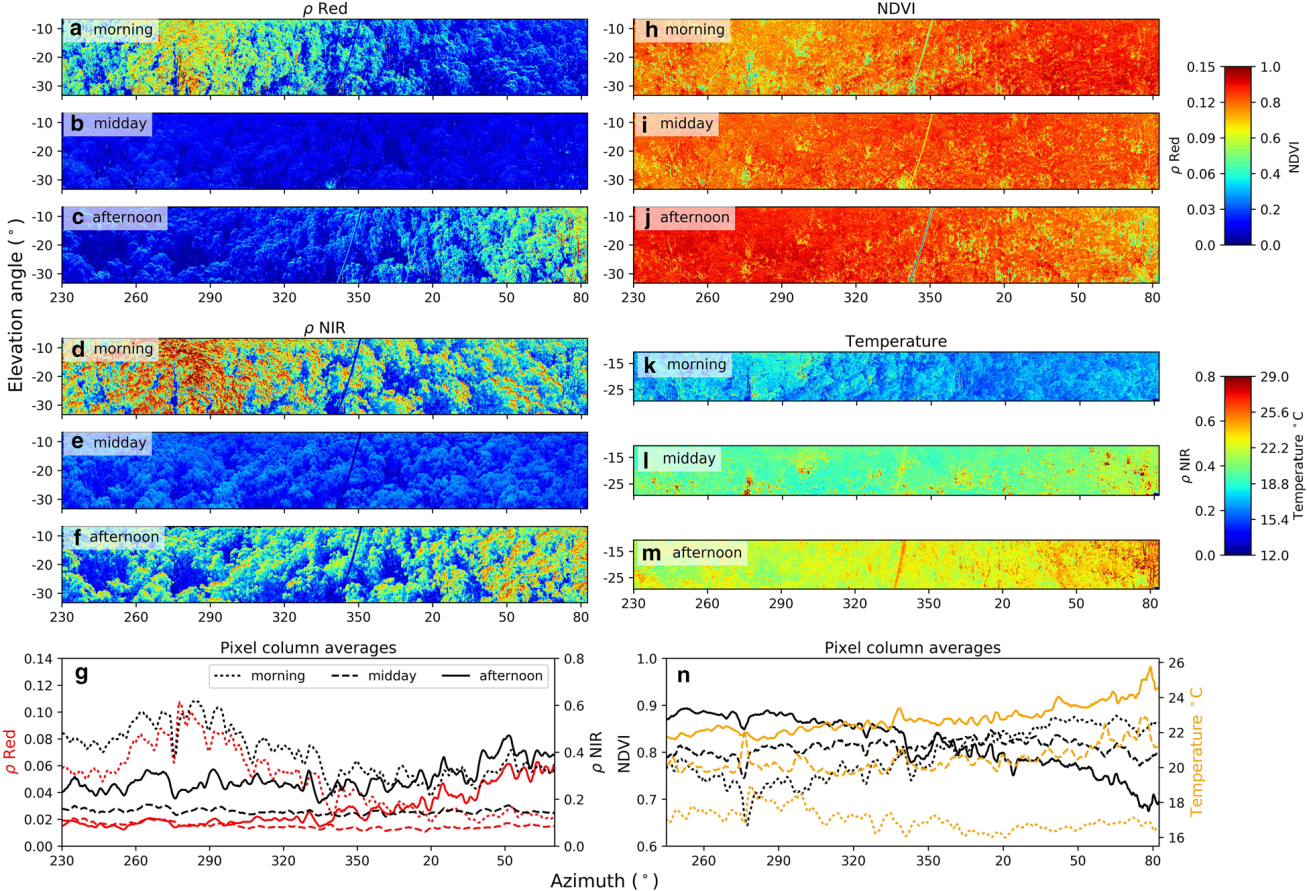
Diurnal variation image panoramas at three times of day on Jan 1st, 2018: morning (07:45), midday (12:11, solar noon) and afternoon (16:40) for the − 20° elevation scan angle. Subplots: **a**–**c** red reflectance ‘*ρ* Red’; **d**–**f** NIR reflectance ‘*ρ* NIR’; **h**–**j** NDVI calculated as (*ρ* NIR − *ρ* Red)/(*ρ* NIR + *ρ* Red) [59]; **k**–**m** range corrected canopy temperature; **g**, **h** display the pixel column averages of the panels in each column above. Sun positions for each acquisition (azimuth, elevation): morning (98°, 31°), midday (0°, 77°), afternoon (− 97°, 31°). Time of day in UTC + 10

The visible wavelengths are more strongly affected by different viewing geometries than the NIR spectrum due to lower amounts of multiple scattering from strong pigment absorption (Fig. 6a–g). These differences are carried through to NDVI, the normalised difference between red and NIR reflectance, which creates a gradient along the azimuthal axis in the morning and afternoon acquisitions (Fig. 6h, j, n). The gradient is also apparent in the thermal imagery caused by the changing proportion of sunlit and shaded canopy. However, unlike the visible and NIR reflectance, the scene temperature magnitude undergoes a large change between the diurnal acquisitions as the temperature continually increases and largely decouples from incoming solar radiation in the afternoon during clear sky conditions (Fig. 6k–n).

**Fig. 7:**
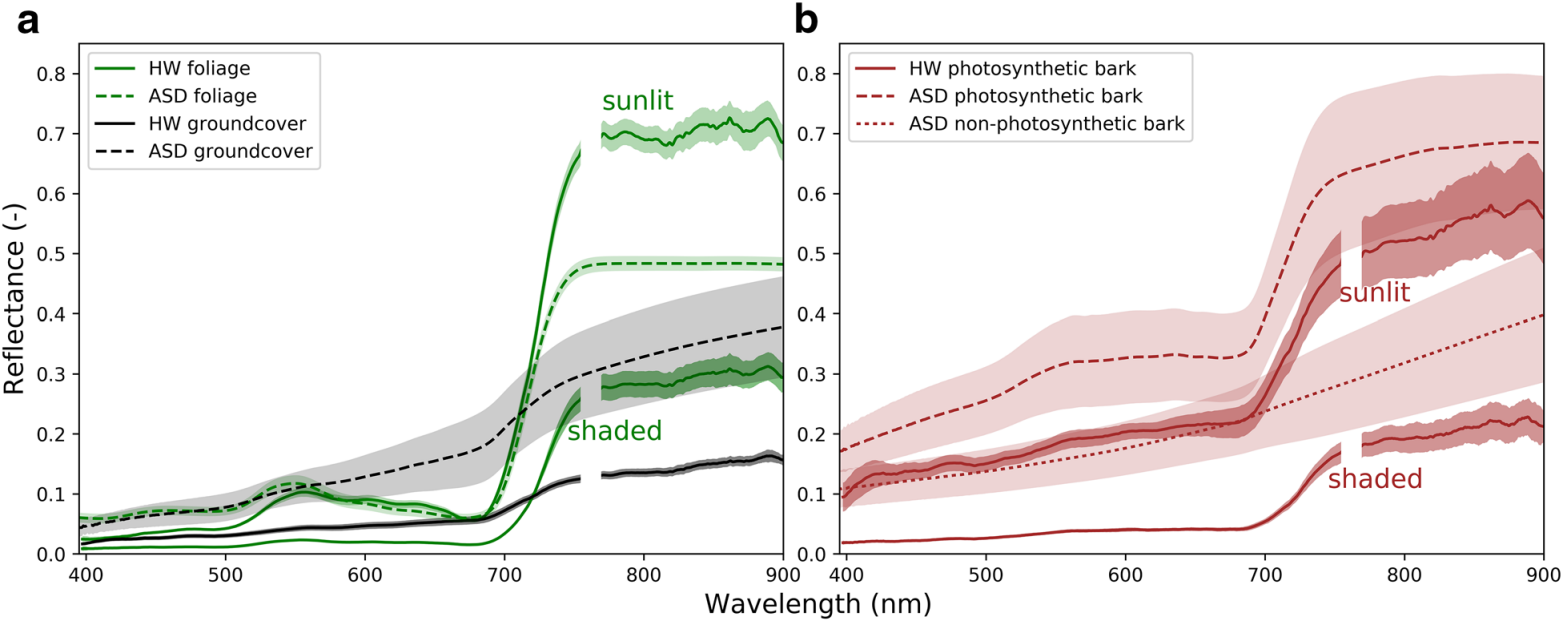
End-member spectral plots for the HLS Headwall ‘HW’ hyperspectral system and an ‘ASD’ Field Spec 3 spectrometer (ASD Inc.; Boulder, CO) for foliage and groundcover (**a**) and bark (**b**). The HW provides hemispherical-conical reflectance factors from multiple sensor and sun geometries, whereas the ASD provides a fixed sensor-illumination source geometry providing biconical reflectance factors [62]. One standard deviation around the mean is shaded. The region around the HW O_2_A absorption band between 755 and 770 nm has been removed for aesthetics due to a misalignment of the two hyperspectral sensors less than one band (< 1 nm) in magnitude

The high spatial resolution of the system enables specific scene end-members such as foliage and bark to be resolved with ease, in both the thermal and hyperspectral imagery. The pixel size from the thermal and hyperspectral imagers ranges from 3 cm for top-of-canopy pixels at the oblique viewing angles to less than 15 cm at a range of around 300 m for the shallow elevation angles near the horizon. Such a fine resolution enables groups of pixels to be identified on a single branch for example, as well as a clear delineation of sunlit and shaded regions to extract the full spectral profile (Fig. 7). The end-members extracted from the HLS imagery show good agreement with spectral features from pure spectral collected with the hand-held ASD spectrometer (Fig. 7). For example, at Tumbarumba we see photosynthetically active bark, which displays the characteristic red edge inflection of foliage pixels (Fig. 7b).

**Fig. 8:**
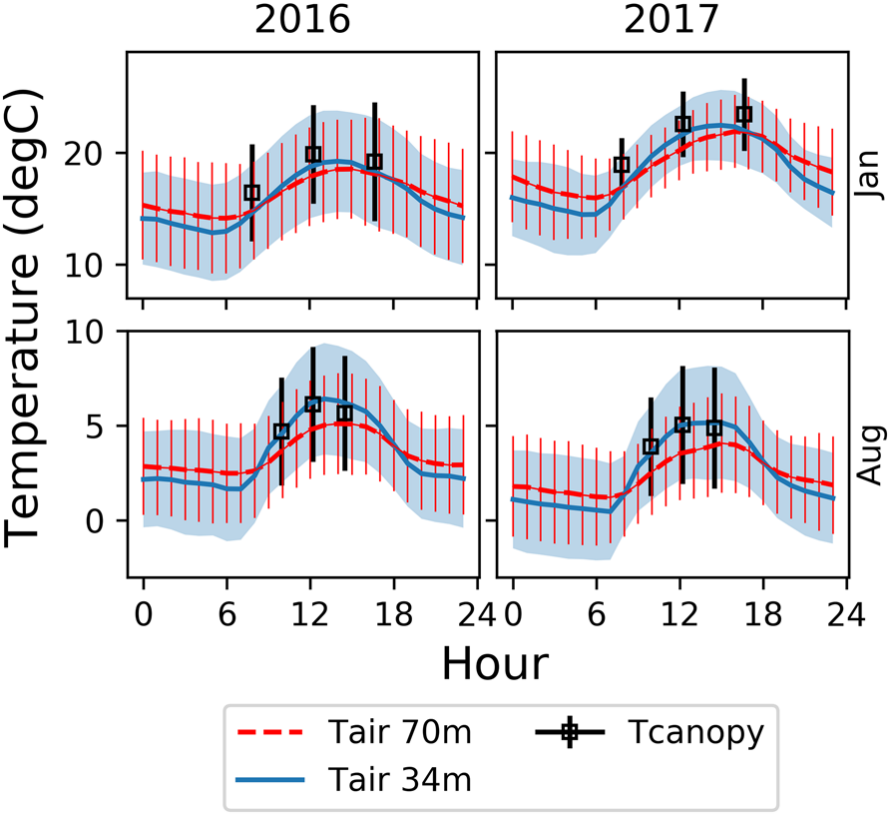
Monthly diel (24 h) temperature plots for January and August 2016 and 2017. The blue line denotes air temperature at 34 m, the red line denotes air temperature at 70 m, and black squares denote canopy temperature from the thermal camera at the three sun elevation reference angles. Error bars (1SD) are overlayed. Air temperature was measured with thermocouples mounted under radiation shields. Ground cover pixels were masked out of the thermal imagery using the co-registered TLS derived canopy mask. The other calendar months were omitted for figure clarity

The canopy temperature from the thermal imagery closely tracks the diurnal behaviour of the air temperature for the subset of the two-year observational period shown in Fig. 8. In general, the median canopy temperature more closely tracks air temperature at 34 m than air temperature at 70 m, reflecting a stronger coupling in the canopy than above as expected. The highest canopy to air temperature differences were observed in summer for both 2016 and 2017, with the canopy temperature warmer than both measured air temperatures indicative of a lagged acclimation to higher air temperatures.

**Fig. 9:**
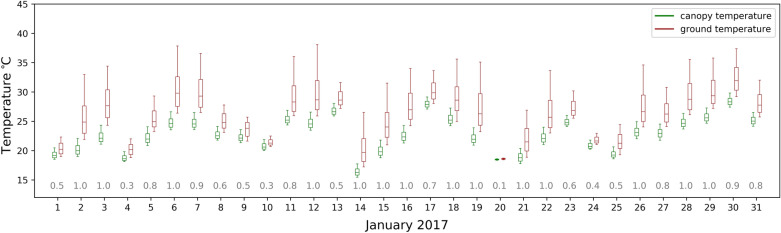
Temperature box plots for canopy (green) and ground (red-brown) for January, 2017. Thermal scans are for solar noon restricted to the symmetrical − 40° to 40° view azimuth range. The boxplot markers denote the 2nd, 25th, 50th, 75th and 98th percentiles. The − 32.5° elevation scan angle was used. Clearness index values for each day are in the grey text (values between 0 and 1), calculated as the ratio of shortwave radiation observed to a theoretical clear sky flux. Low clearness index values denote cloudy days

The ground surface was typically warmer than the canopy during the solar noon thermal imagery acquisitions due to evaporative cooling of canopy (Fig. 9). The maximum ground temperatures were much warmer than the canopy, with larger differences observed under clear sky conditions with more than 10 °C differences in maximum temperature. The solar noon acquisitions during January was the time of year with maximal difference between canopy and ground temperature as opposed to the morning and afternoon reference angle acquisitions, as solar noon coincides with the maximum sunlit ground area from a maximum sun elevation angle (data not shown). The very small temperature range (< 2 °C) on 20th January was a day with heavy fog and rain (clearness index value 0.1) and could arguably be filtered from the analysis depending on its aim.

**Fig. 10:**
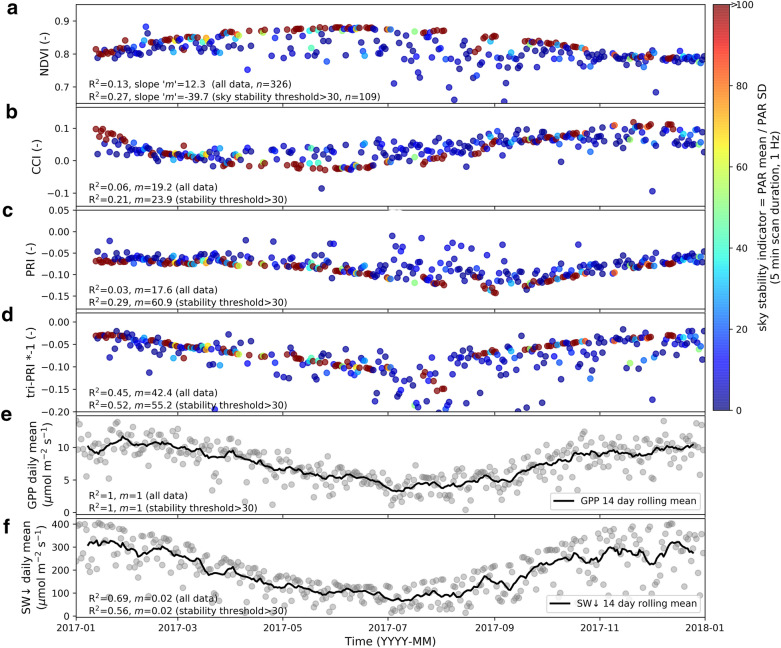
A 2017 time series of vegetation indices **a**–**d**, daily mean GPP and shortwave down-welling radiation ‘SW↓’ (**d** and **e**, respectively). Vegetation indices are calculated from solar noon scans with an 80° symmetric FOV centred on 0° azimuth. Vegetation indices are coloured by the sky stability indicator. Linear regression R^2^ and slope values use daily observations correlated with daily GPP and are inserted in each subplot. The first row of the R^2^ and slope values use all valid data (n = 326), and the second line uses the filtered data with a sky stability threshold > 30 (n = 109). All p-values < 0.001. Some outliers are outside the y-axis bounds to maintain figure clarity

The triangular photochemical reflectance index ‘tri-PRI’ was the spectral indicator that tracked daily averaged GPP the closest over the full 12 month period (R^2^ = 0.45 and 0.52 for all data and filtered unstable sky conditions, respectively), outperforming NDVI, CCI, and PRI (Fig. 10). The vegetation index scatter decreased and all R^2^ values increased as unstable sky conditions were filtered out to reveal a smooth trend with distinct seasonal patterns. A sky stability indicator value of 30, representing mean 1 Hz PAR being greater than 30 times its standard deviation over the course of each 5 min scan, coincided with R^2^ values largely flattening out (data not shown). This led to approximately one third of scans remaining under ‘stable’ sky conditions. NDVI was negatively correlated with GPP once the unsteady sky conditions were filtered out.

## Discussion

### The importance of multi-sensor, -temporal, and -angular data streams

Multi-sensor data streams underpin and greatly enhance the value of proximal sensing systems. They are used in a broad spectrum of applications from data corrections, filtering and quality assurance, through to increasing ecophysiological understanding from multiple lines of evidence. In addition to previously mentioned integrated proximal systems at flux tower sites, plant phenotyping is a main driver of technical development, evidenced by static tower systems [1] or mobile systems carrying multiple payloads [6, 16, 68]. Such systems facilitate research into long-term ecosystem dynamics, monitoring environmental change and ecosystem resilience.

Ancillary measurement streams are also required for thermal imagery atmospheric transmission corrections, including sky temperature, relative humidity and air temperature. These are common data streams from flux towers [5]. The path length from the sensor to the canopy is also a key parameter for thermal corrections especially for a multi-angular system with a greatly varying path length to the target (10’s to 100’s meters). This type of ranging data can be acquired from photogrammetric techniques [5] or LiDAR techniques as used in this study. Kim et al. [44] used thermocouples attached to conifer needles to determine that the FLIR thermal camera-derived leaf temperature was slightly underestimated at low ambient temperatures (< 5 °C) and developed a correction factor. They also used ancillary flux tower meteorological data to determine the degree of leaf temperature coupling with the atmosphere and explained its main drivers, which are key functional parameters related to photosynthetic performance [9, 31].

The enhanced spectral resolution of THEMS which combines hyperspectral bands in the VNIR region with a thermal band paves the way for measuring multiple ecosystem function indicators. In particular, the full VNIR spectrum may enable improved detection of subtle phenological events in the evergreen Tumbarumba canopy such as leaf emergence and senescence, which is typically a co-occurring event at the site [42].

Spectrally sensed phenological shifts can also be linked to corresponding changes leaf photosynthetic capacity [19]. GPP at Tumbarumba is mainly driven by incoming radiation [66]. Interestingly, NDVI was anti-correlated with mean daily GPP over the course of a 1 year time period after filtering out unstable sky conditions (Fig. 10). Two possible explanatory factors are: (i) there was slightly more overall greenness in winter due to the grassy understorey greening up with increased winter rainfall (data not shown), and (ii) there is a higher proportion of the spectral signal coming from the canopy versus the ground layer in winter due to lower sun elevation angles, where the ground layer also comprises bare earth and other non-photosynthetic material. tri-PRI tracked the GPP seasonality and outperformed CCI, and PRI (Fig. 10), which were developed in crops and northern hemisphere species [21, 22], whereas tri-PRI was developed from leaf-level eucalypt spectral data at Tumbarumba albeit from a single time of year [70]. Similar to PRI, tri-PRI over the full year was likely to be largely driven by constitutive pigment pools changes, where evergreen eucalypt species are known to increase their carotenoid concentration and reduce chlorophyll for ‘cold temperature hardening’ during winter stress periods [27]. More work is required to investigate the canopy scale link across seasons between indices like PRI and short-term light use efficiency changes, especially at Tumbarumba. Linking photosynthetic seasonality across multiple lines of observation including fluxes, leaf pigment content, and non-destructive spectral measures will bridge spatio-temporal domains and continue to fill ecosystem knowledge gaps [13, 50, 56].

The high spatial resolution of both the thermal and VNIR HLS sensors in THEMS enables pinpointing canopy dynamics in space and time. For example, individual trees and sections of crowns are easily identifiable in the imagery and thus are more directly relatable to crown and leaf level measurements. At a fine spatial scale, pinpointing canopy dynamics can facilitate spectral scaling of independently measured plant function processes such as water use and transpiration from sap flow velocity measurements and above-ground growth from dendrometers or LiDAR data. The enhanced spatially resolved capability can also assist with microclimatic measurements to further our mechanistic understanding of the impact of topography and vegetation structure [72]. The advantage of high spatial resolution imagery also extends to aiding spectral unmixing techniques from coarse resolution sensors by resolving specific scene end-members (Figs. 4, 7) such as; separating canopy from ground cover or background, sunlit from shaded areas, and photosynthetic from non-photosynthetic material [4]. This is critical for isolating sunlit regions of the canopy for remotely sensed dynamic physiological indicators [32]. Flowering events may also be detected from increased spatial and spectral resolution (data not shown) where the full spectrum of pure end-members may be used to develop retrieval algorithms using tailored spectral bands. The combined spectral sensing capability of thermal and hyperspectral bands provides the ability to probe regional to global remote sensing driven retrieval methodologies of carbon, water and energy fluxes [3, 17] critically using the same spectral input data streams albeit at different scales. Specifically, methodological steps and underlying assumptions can be tested from scarce long-term, high spatial and temporal resolution imagery of reflectance and temperature coincident with flux tower observations.

A multi-angular and large field-of-view (FOV) measurement capability is essential to bridge spatial scales between sensing platforms. The Pan-Tilt Unit (PTU) is a critical feature of THEMS that enables multi-angular observations from a fixed point both in azimuth and elevation viewing directions, as opposed to viewing the same object from multiple directions. This capability permits an area comparable to the flux tower footprint to be measured under typical daytime turbulent conditions [18, 45, 66]. Two key further advantages of a PTU coupled with a narrow FOV sensor over a downward pointing hemispherical FOV or wide-angle sensor are: (i) the view-geometry of a satellite or above-canopy platform can be more closely matched, and (ii) better characterisation of the site BRDF to verify modelled products. Specifically, multi-angular observations over homogeneous land cover permit application of BRDF modelling approaches to separate physiological from structural and view-geometry effects across temporal scales from hours to seasons where the sun angle changes significantly [33]. These types of observations are increasingly relevant as BRDF models and corrections are an area of ongoing development especially at high spatial resolutions and at canopy scales with complex architecture [15, 24, 38, 73].

### THEMS limitations and challenges

The increased spatial-, spectral-, and temporal-resolution of THEMS presents a data storage trade-off in a resource constrained environment. Operating the system to collect hourly acquisitions during daylight hours in summer would equate to nearly 0.5 TB of data per day. As the system is intended for long-term autonomous use the trade-off we employed was to reduce the number of daily acquisitions to three, coinciding with predefined reference sun elevation angles (morning, solar noon, and afternoon). A potential solution to this big data problem could be to filter data on-the-fly to produce data products if specific applications were known a priori. However, the system is an exploratory research tool with the full system potential yet to be evaluated. High-speed internet via satellite or cellular telephone network connection is also a potential future solution but not immediately feasible at the site. A recent NAS upgrade to 64 TB of storage at the site has made on-going hourly acquisitions feasible.

The high spatial resolution of THEMS combined with a tall canopy forest with complex canopy architecture presents a challenge for the accuracy of reflectance-based indices computed by normalising radiance against an above-canopy hemispherical irradiance reaching a horizontal plane, as opposed to irradiance reaching the target surface. Mõttus et al. [53] found the magnitude of the physiological variation of the Photochemical Reflectance Index (PRI), derived as a reflectance ratio in the green wavelengths, was equal to the variation induced from normalising against a diffuse light spectrum as opposed to a direct light spectrum. This effect is particularly important for shadowed pixels mainly illuminated by diffuse radiation, and exacerbated by highly clumped and vertically inclined foliage of Eucalypts [37]. Reflectance standard panels spatially distributed in the sensor FOV would be a potential solution to this problem but highly impractical especially for multi-angular sensors with a large areal coverage (> 1 ha). Additionally, 3D radiative transfer modelling of such ecosystems would provide a potential avenue to characterise the BRDF contribution under a wide variety of simulation conditions [54, 69]. However, this approach requires a significant amount expertise, time investment and computer modelling capability [11].

A key methodological assumption was a constant 0.95 emissivity for all scene components. Canopy emissivity is a function of directional and volumetric properties [40]. Thus, canopy emissivity varies as a function of scale with implications for energy balance modelling for instance. Individual leaves may have an emissivity around 0.95 whereas multiple leaves in a single pixel may have an emissivity closer to 0.98–0.99 due to multiple reflections and shadowing [26, 28, 39]. A single pixel is comparable to the leaf size at oblique viewing angles at the site and thus a 0.95 emissivity value is appropriate. Whereas a higher emissivity value may be more appropriate for pixels near the horizon [5].

Multiple physiological, structural and meteorological factors influence leaf surface temperature [49]. The largest thermal imagery temperature gradients were caused by (i) the sun angle creating so-called hot-spots and dark-spots driven by changing shadow fraction (Figs. 4, 6), and by (ii) the target type where bare soil and ground displayed much warmer temperatures than the canopy under direct illumination conditions (Fig. 5). These ground temperatures may be an slight underestimate due a lower soil emissivity of 0.9–0.92 perhaps being more appropriate [39]. More sophisticated methods of modelling target emissivity as a function of distance to the canopy and sensor characteristics is an area for further development.

The 5-min acquisition time for a single panoramic scan covering 240° in azimuth presents challenges under changing illumination conditions caused by cloud cover. The original system design collected an irradiance measurement at the beginning and end of each elevation angle acquisition. A recent system modification saw irradiance measurements collected every few seconds, where in both cases a linear change in sky conditions is assumed between measurements. However, under cloudy conditions lighting can change rapidly thus potentially limiting the usefulness of scans. A further challenge presents itself when relating this data to the carbon, water, and energy fluxes measured by the co-located tower instrumentation, which is aggregated to a one hourly time interval to capture the range of eddies at this tall canopy site [46]. In this case it is imperative that the sky conditions during the five minute THEMS acquisitions are representative of the full hourly averaging period, as sunlight is the main driver of canopy and ecosystem function [66]. We recommend the collection one second frequency PAR data so that the five minute and one hourly THEMS measurement windows can be compared with aggregated flux tower data and checked for consistency. The degree of data filtering strictness can be user-determined and led by the application of interest.

Suggested developments to enhance the monitoring system’s capability would focus on incorporating additional measurement streams. For example, the shortwave infrared (SWIR) spectral region is seldom sensed by proximal systems yet is a promising tool for monitoring live fuel moisture content (LFMC), especially relevant for bushfire-related applications [36, 64, 71]. A SIF measurement capability from a co-located high spectral resolution NIR spectrometer would open up the possibility to investigate multiple avenues of photosynthetic activity and plant stress [56], and has recently been installed at the site. Additionally, a PTU upgrade was recently made to accommodate a heavier payload so that viewing angles closer to nadir can be measured, which is a limitation of the original system design presented here. Lastly, a co-located aerosol monitoring station [35] is recommended to reduce uncertainties for satellite platform comparisons of radiance, reflectance, and temperature; and was installed 15 km from the flux tower site in July, 2019.

## Conclusions

This manuscript presents the technical details of the proximal observation system ‘THEMS’ for collecting long-term, unattended, continuous and multi-angular thermal and hyperspectral imagery at a highly instrumented flux tower site. The high spatial resolution permits scaling along the leaf-to-canopy continuum, enabling direct comparison with leaf level measurements through to scales comparable with the flux tower and satellite footprints. The multi-purpose system is intended to provide unprecedented spatio-temporal ecophysiological insight and to underpin upscaling of remotely sensed dynamic ecosystem water, CO_2_, and energy exchange processes.

## Supplementary information

**Additional file 1: Figure S1.** A screengrab of the SuperTHEMS and THEMS GUI executed as LabVIEW virtual instruments. The ‘Main’ tabs are depicted, with more functionality in the separate tabs (not shown), especially for THEMS (12 tabs total). **Figure S2.** Monthly diel temperature plots for 2016 and 2017. The blue line denotes 34 m air temperature, the red line denotes 70 m air temperature, and black squares denote canopy temperature derived from the thermal camera at the three sun elevation reference angles. Error bars (1SD) are overlayed.

## Data Availability

The datasets during and/or analysed during the current study available from the corresponding author on reasonable request.
