# Supplementary material for: THEMS: an automated thermal and hyperspectral proximal sensing system for canopy reflectance, radiance and temperature
Source: Plant Methods. 2020 Jul 31;16:105. doi: 10.1186/s13007-020-00646-w (PMC7395347; doi:10.1186/s13007-020-00646-w)
Supplement: Supplementary file 1 — Additional file 1: Figure S1. A screengrab of the SuperTHEMS and THEMS GUI executed as LabVIEW virtual instruments. The ‘Main’ tabs are depicted, with more functionality in the separate tabs (not shown), especially for THEMS (12 tabs total). Figure S2. Monthly diel temperature plots for 2016 and 2017. The blue line denotes 34 m air temperature, the red line denotes 70 m air temperature, and black squares denote canopy temperature derived from the thermal camera at the three sun elevation reference angles. Error bars (1SD) are overlayed. [file 13007_2020_646_MOESM1_ESM.docx]

**Additional Material**

Manuscript title: *An automated thermal and hyperspectral proximal sensing system for canopy reflectance, radiance and temperature: THEMS*


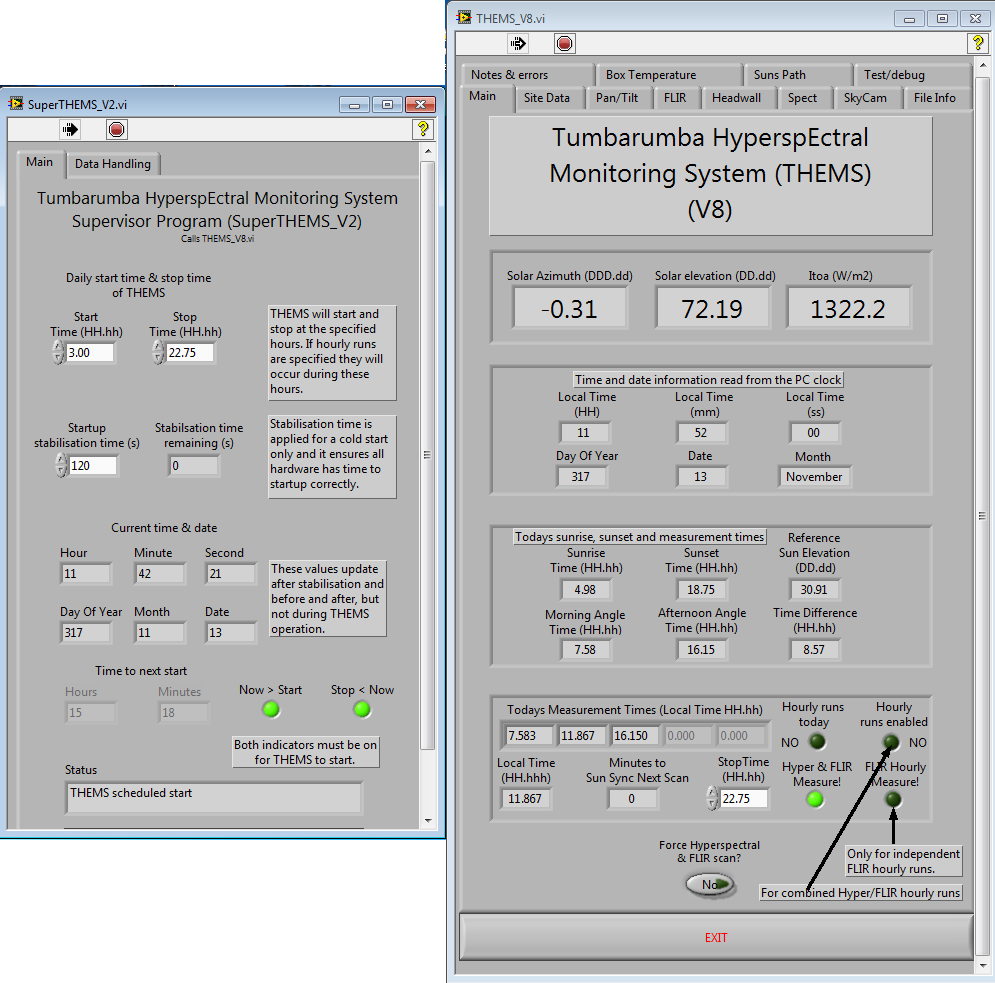


**Figure S1.** A screengrab of the SuperTHEMS and THEMS GUI executed as LabVIEW virtual instruments. The ‘Main’ tabs are depicted, with more functionality in the separate tabs (not shown), especially for THEMS (12 tabs total).


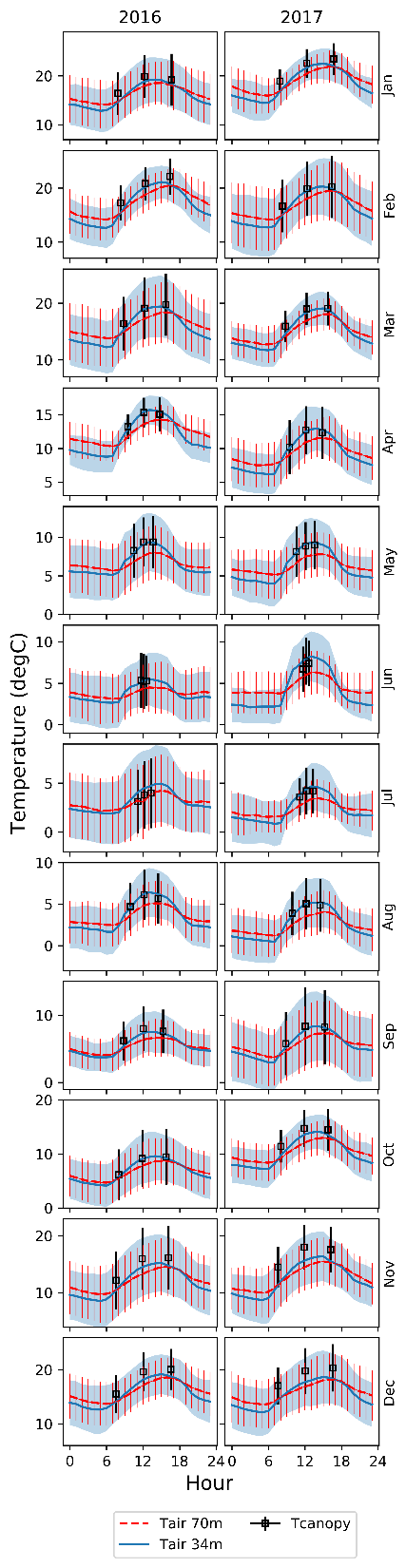


**Figure S2.** Monthly diel temperature plots for 2016 and 2017. The blue line denotes 34 m air temperature, the red line denotes 70 m air temperature, and black squares denote canopy temperature derived from the thermal camera at the three sun elevation reference angles. Error bars (1SD) are overlayed.
